# Machine Learning Techniques for Arterial Pressure Waveform Analysis

**DOI:** 10.3390/jpm3020082

**Published:** 2013-05-02

**Authors:** Vânia G. Almeida, João Vieira, Pedro Santos, Tânia Pereira, H. Catarina Pereira, Carlos Correia, Mariano Pego, João Cardoso

**Affiliations:** 1 Instrumentation Center, Physics Department, University of Coimbra, Rua Larga, Coimbra 3004-516, Portugal; 2 ISA-Intelligent Sensing Anywhere, Coimbra 3030-320, Portugal; 3 Cardiology Department, Coimbra Hospital and University Centre (CHUC), Coimbra 3000-075, Portugal

**Keywords:** pulse wave analysis, arterial pressure waveform, machine learning, arterial stiffness, augmentation index

## Abstract

The Arterial Pressure Waveform (APW) can provide essential information about arterial wall integrity and arterial stiffness. Most of APW analysis frameworks individually process each hemodynamic parameter and do not evaluate inter-dependencies in the overall pulse morphology. The key contribution of this work is the use of machine learning algorithms to deal with vectorized features extracted from APW. With this purpose, we follow a five-step evaluation methodology: (1) a custom-designed, non-invasive, electromechanical device was used in the data collection from 50 subjects; (2) the acquired position and amplitude of onset, Systolic Peak (SP), Point of Inflection (Pi) and Dicrotic Wave (DW) were used for the computation of some morphological attributes; (3) pre-processing work on the datasets was performed in order to reduce the number of input features and increase the model accuracy by selecting the most relevant ones; (4) classification of the dataset was carried out using four different machine learning algorithms: Random Forest, BayesNet (probabilistic), J48 (decision tree) and RIPPER (rule-based induction); and (5) we evaluate the trained models, using the majority-voting system, comparatively to the respective calculated Augmentation Index (AIx). Classification algorithms have been proved to be efficient, in particular Random Forest has shown good accuracy (96.95%) and high area under the curve (AUC) of a Receiver Operating Characteristic (ROC) curve (0.961). Finally, during validation tests, a correlation between high risk labels, retrieved from the multi-parametric approach, and positive AIx values was verified. This approach gives allowance for designing new hemodynamic morphology vectors and techniques for multiple APW analysis, thus improving the arterial pulse understanding, especially when compared to traditional single-parameter analysis, where the failure in one parameter measurement component, such as Pi, can jeopardize the whole evaluation.

## 1. Introduction

The development of feasible surveillance methods to assess patterns and trends associated with major cardiovascular diseases improves the prevention and identification of cardiovascular risk factors, as well as the development of new strategies for better arterial pulse analysis.

The assumption that arterial stiffness is a marker of vascular disease and a risk factor for cardiac mortality has gained support over the last years, with several studies documenting its prognostic importance [1,2]. In adults, the relationship with other physiological conditions, such as hypertension, a highly prevalent cardiovascular risk factor worldwide, has been established by several authors [3,4], who emphasize the contribution of pulsatile pressure and vascular resistance [5].

The pulse wave analysis is a non-invasive method based upon the analysis of Arterial Pressure Waveform (APW) components that can accurately deliver information about the cardiovascular status [6]. The growing interest for pulse wave analysis-based devices can shift the paradigm in the risk factor management for cardiovascular diseases. APW is determined by the interaction of the heart blood pumping and the arterial tree, with its multiple vessels, and contains physiological information concealed by its morphology. Many factors determine its morphology, the most influential being: the ejected heart blood volume, the aortic valve closure and the elasticity and the integrity of the blood vessels.

The development of non-invasive methods for this particular purpose remains an interesting topic for many researchers. Non-invasive APW can be obtained by applanation tonometry, where a superficial artery is flattened against a bone structure [7]. This technique is only possible in peripheral arteries to avoid the effect of the subcutaneous tissues between the tonometer and the artery. The disadvantages of the tonometer include its sensitivity to sensor placement that should be properly immobilized upon the artery of interest, as well as the movement artifacts that may lead to signal distortions, during the acquisition [7,8]. Diameter waveform measurements through ultrasound techniques can provide a valid alternative [9], but time resolution limits its use due to the accomplished low frame rate. The use of piezoelectric (PZ) sensors in APW measurements is an interesting solution that has been reported by several authors [10,11]. In a previous work, a custom-designed probe for carotid APW analysis has already been developed [12]. Other works, related to the management of neurosurgical disorders, have also studied the waveform morphology of other physiological signals, such as the intracranial pressure through the analysis of peak locations [13,14] and the temporal dependency between successive pulses [15].

Amongst the most studied hemodynamic parameters, the Pulse Wave Velocity (PWV) and Augmentation Index (AIx) [2,16], are frequently described as independent predictors of cardiovascular risk [1]. There is a number of commercialized devices that estimate arterial stiffness PWV or AIx values [17], such as Complior (Alam Medical) [18] and Sphygmocor (AtCor Medical) [19]. The combined analysis of other features taken from the APW could be an interesting topic that can enhance the pulse wave comprehension. However, the exploration of a high number of parameters over this bio-signal requires complex processing algorithms, such as those used in machine learning techniques that may cope with its inherent variability, over different subjects and acquisition conditions, and still provide reliable and conclusive information.

Computer-aided diagnosis methodologies based on data mining and machine learning techniques have been used in many biomedical applications during the last years [20,21,22,23]. These techniques could contribute to the development of clinical decision-making approaches using information taken from different measurements setups, overcoming the limitations in the management of complex and unstructured data [24]. In order to overcome errors resulting from single classifier analysis, multi-classifiers [25] can be used to reduce overall classification errors, incorporating the prediction outcome of each classifier [26].

One of the main goals of our work is to provide a multi-parametric approach capable of retrieving important information from arterial blood pulse waveform, which has not yet been revealed. The combination of carefully selected APW features should significantly improve the arterial blood pulse understanding, especially if compared with the traditional single parameter analysis, where the failure in determining one component, such as the reflected waveform component, can jeopardize the whole assessment. The application of techniques, for predictive value analysis and selection, allows the increase of accuracy in the classification by the selection of the most discriminating features. The current paper is organized as follows: the dataset and data processing are described in section II, experimental results and discussion are presented in sections III and IV, respectively, and conclusions and future work are drawn in the last section.

## 2. Methods

### 2.1. Signal Database

The pulse waveforms used in this study were obtained from 50 subjects divided in three groups, as described below. Data were collected with approval by the ethical committees of the Coimbra Hospital and University Centre (CHUC), Portugal, with informed consent.

- Group I is constituted by 20 hypertensive subjects. Hypertension was diagnosed when systolic blood pressure (SBP) ≥ 140 mmHg and/or diastolic blood pressure (DBP) ≥ 90 mmHg, or if the patient was taking anti-hypertensive medication. The data were acquired during hospitalization, but prior to taking any medication. A total of 2,014 stored pulses were analyzed.
- Group II is constituted by 20 healthy volunteers that have no documented history of cardiovascular disorders. The taken data resulted in an overall of 1,959 pulses.
- Group III is constituted by 10 volunteers that will be analyzed during validation procedures. In spite of being young subjects, these individuals present pulse waveform characteristics of individuals that are developing arterial disorders. To accomplish this selection, a preliminary waveform analysis based on the waveform types (that are described in the Section 2.3.2) was performed. The overall data in this group represent 627 pulses.

**Figure 1:**
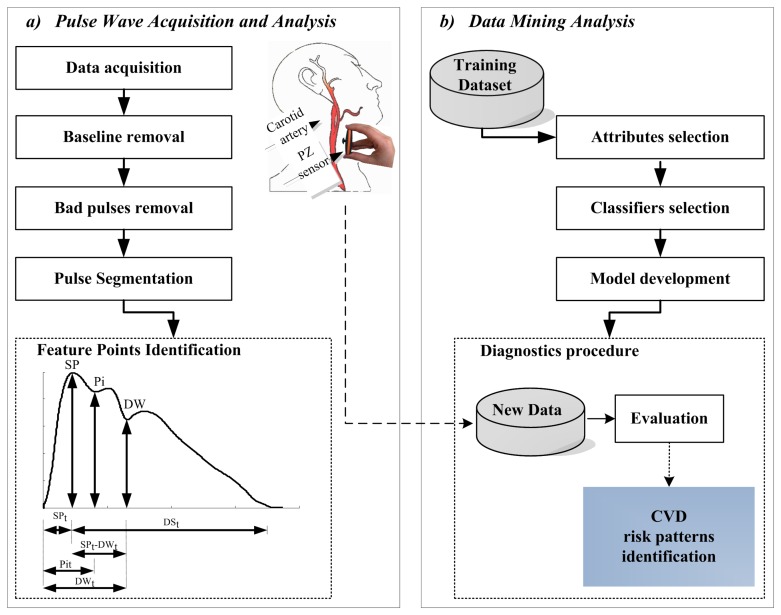
Schematic representation of the overall analysis process: (**a**) Pulse wave acquisition and analysis processes, where some features are identified: Systolic Point (SP), Point of Inflection (Pi) and Dicrotic Wave (DW). These features are used in the computation of: upstroke time (*SP_t_*), time at Pi (*Pi_t_*), time at DW (*DW_t_*), downstroke time (*DS_t_*) and the systolic and the diastolic time difference (*SP_t_* – *DW_t_*); (**b**) major tasks performed during data mining analysis.

APWs were recorded at a sampling rate of 1 kHz with a non-invasive PZ probe developed in a previous work [12], and shown in Figure 1a. The probe is held in place by a neck collar specially developed for carotid measurements. The mechanical interface also plays an important role. This is based on a mushroom-shaped sensor that transmits the distension associated to the pressure wave in such a way that transversal and shear effects are suppressed and only radial applied forces are allowed. Accuracy tests were performed at a bench test, where 1.80% was the maximum Root Mean Square Error (RMSE) introduced by the electronic circuits and by the mechanical interface.

The pressure values (systolic - SBP, diastolic - DBP) and heart rate (HR) values were measured by a commercially available device (OMRON M6 Comfort) and included in the dataset. Age, gender, smoker habits, height, weight and Body Mass Index (BMI) were also registered for all subjects. Demographic data were compared among groups by using one-way analysis of variance (one-way ANOVA).

### 2.2. Introduction of the Framework

The block diagram of the framework for the analysis used along this work is represented in Figure 1. The two major steps after data acquisition include: pulse wave analysis and data mining techniques application. Pulse-by-pulse analysis was conducted using an algorithm developed in a previous work [27] based on the first order derivative of the APW. The quality of the results was statistically quantified both in time and amplitude. An error rate of 4.20% was observed in time measurements and of 2.68% for amplitude measurements. This algorithm is also able to remove the baseline, to make pulse-by-pulse segmentation and to flag anomalous beats, based on a set of subject morphological features which includes: pulse amplitude and pulse width variations. Morphological variations can occur during the monitoring, being responsible for the detected anomalous feature points. This might occur from the combination of several conditions, such as: patient movements, respiration rate, noise and artifacts associated with subjects' physiological variability and even with the acquisition device.

This methodology was developed for the database exploration of hemodynamic features from APW morphology that hold essential information about arterial wall integrity. This information also establishes important indices about the arterial stiffness assessment that show the alterations in the mechanical properties of the arteries. The data mining methodology is based on two steps: in first, the most relevant features are selected and then four classification algorithms are applied based on accuracy measurements; finally, the model is validated with reference to the well-established AIx parameter.

### 2.3. Pulse Wave Analysis

Pulse wave analysis relies on a clean APW, in which the prominent morphological features are reliably identified. The main determinants of APW morphology are: Systolic Peak (SP), Point of Inflection (Pi) (caused by reflected wave) and Dicrotic Wave (DW). SP results from the blood ejected from the left ventricle, while wave reflections occur due to the action of the forward wave traveling along the arterial tree and a backward wave returning towards the heart from the reflection sites [2]. These waves superimpose on the forward wave, thereby originating a visible change in the APW profile and contributing to the highlighted Pi. The DW occurs when the aortic valve closes moving a small portion of the ejected blood back to the left ventricle. The prominent point locations are schematically represented in Figure 1a.

In this work, several ways of retrieving hemodynamic features from APW measurements were explored and are described as follows:

#### 2.3.1. Time and Amplitude Intervals

Six APW intervals are considered for each pulse, as depicted in Figure 1a. These parameters include the time intervals: the time to the systolic peak (SP*_t_*), the time to the point of inflection (Pi*_t_*) and the time to the dicrotic wave (DW*_t_*); and, the amplitude considerations: the height at the systolic peak (SP*_a_*), the height at the inflection point (Pi*_a_*) and the height at the dicrotic wave (DW*_a_*).

#### 2.3.2. Ratios and Indices

Although time and amplitude parameters are effective, they lack the ability to discriminate patterns within the whole pulse morphology. Six ratios are computed according to Table 1 based on time and amplitude intervals described above.

**Table 1:** Ratios and indices used in the multi-parametric approach.

**Attribute**	**Description**	**Units**
$R1=\frac{SP_{t}}{T}$	Upstroke time ratio	[-]
$R2=\frac{SP_{t}-DW_{t}}{T}$	Downstroke time ratio calculated between the systolic and the diastolic waves	[-]
$R3=\frac{DS_{t}}{SP_{t}}$	Time ratio calculated between the global downstroke time (*DS_t_*) and the upstroke time (*SP_t_*)	[-]
$R4=\frac{DW_{a}}{SP_{a}}$	Time ratio between the dicrotic and the systolic heights (*SP_a_*)	[-]
*R*5 = |*SP_a_* – *Pi_a_*|	Amplitude difference module between the systolic and the inflection points	[V]
$R6=\frac{Pi_{a}}{SP_{a}}$	The systolic and the inflection points amplitude ratio	[-]

The well-established AIx, which measures the augmented component of pressure is computed according Equation 1 denned by Murgo *et al.* [28], where A is the normalized amplitude.

(1) $$\text{AIx}(\%)=\pm \frac{SP_{a}-Pi_{a}}{A}\times 100$$

The key issue in the AIx computation is related with Pi identification after or before the systolic wave front. Negative values are used to distinguish pulses where the Pi arrives after the systole (*Pi_a_* – *SP_a_*), from positive values that occur when the Pi occurs before the systolic peak (*SP_a_* – *Pi_a_*), causing a pressure increase. Depending on the AIx value (positive or negative) the pulse wave type is defined as follows: when a negative value occurs the pulse is type C (characteristic of healthy subjects) and when a positive value occurs the pulse is type A (characteristic of subjects suffering from arterial stiffness).

#### 2.3.3. Pulse Variance

Some statistical measurements were performed to assess the variance associated to pulse morphology and prominent points locations, in time and amplitude. The Root Mean Square Successive Difference (RMSSD) was computed for all variables; and the overall list includes: RMSSD*_SPt_*, RMSSD*_SPa_*, RMSSD*_Pit_*, RMSSD*_Pia_*, RMSSD*_DWt_* and RMSSD*_DWa_*. The pulse morphology variability was assessed using the Root Mean Square Error (RMSE) technique, where the mean pulse was taken as a reference. The Full Width at Half Maximum (FWHM) was also computed for all pulses. The attributes are described in Table 2.

**Table 2:** Pulse variance attributes.

**Pulse Variance**	**Description**	**Units**
RMSSD *_x_* $\sqrt{\frac{\sum_{i=1}^{n-1}{\left(x_{i+1}-x_{i}\right)}^{2}}{n-1}}$	Root mean square of successive differences (RMSSD), where *x* is: SP time (SP*_t_*) SP amplitude (SP*_a_*) DW time (DW*_t_*) DW amplitude (DW*_a_*) Pi time (Pi*_t_*) Pi amplitude (Pi*_a_*)	[%]
RMSE $\sqrt{\frac{\sum_{i=1}^{n-1}{\left(x_{1,i}-x_{2,1}\right)}^{2}}{n}}$	Root-mean-square error (RMSE) (measured in reference to the average pulse)	[%]
FWHM	Full Width at Half Maximum	[%]

### 2.4. Data Mining Techniques

The major motivation of this work was the development of a method to deal with multiple parameters from APW analysis. The application of machine learning algorithms facilitate the learning of a system, from the data at which it is exposed to. That data can include several groups, according to the complexity of the system. Once trained, the system is able to predict a result based on the internal rules previously established, using a set of new inputs. This could bring an interesting impact to the traditional cardiovascular tools for risk prediction. The tools implemented in this work include attribute selection and classifier models evaluation, as depicted in Figure 1b. APW analysis was performed using Matlab 2009a ©, where an output *.arff* file is produced and loaded into the Weka-3-6-5 package [29]. The Weka system was selected as the tool for data analysis due to its efficiency, versatility and affordability.

#### 2.4.1. Attribute Selection

Attribute selection is an important task that allows the improvement of dataset analysis, in a process where the unneeded information is identified due to its low predictive value in the dataset. The identification of redundant and/or irrelevant attributes before the training process allows the optimization of memory space and time machine computing speed requirements, as well as the output understanding. A single-attribute evaluator was performed based on a ranking search method available in the Weka 3.6.5 package—InfoGainAttributeEval [30]. This algorithm is a single-attribute evaluator with a Ranker search method that evaluates the selected attributes, by measuring their information gain, according to the defined classes using a discretization method. The results are ranked according to the average merit (expressed as a number between 0 and 1).

#### 2.4.2. Classification

Data from groups I and II were used, during the training procedure, of the classifiers. Different methods in the machine learning area could be used. Several experiments were made in order to select the most suitable method both from the standpoint of discrimination ability, as well as, from the time spent to make a decision. The evaluation was focused on the following classifiers: J48, Random Forest, BayesNet and JRIP, that are different types of machine learning algorithms and had previous successful application marks in cardiovascular fields [21,31,32]. The specifications of each classification algorithm are described below:

- The J48 is the Weka implementation of C4.5 algorithm, the most popular tree classifier, which was developed by Quinlan (1993). Its implementation is based on a non-backtracking approach. J48 use two heuristic criteria to rank possible tests: the information gain and the default gain ratio. After the building process, each attribute test, along the path (from the root to the leaf) becomes a rule antecedence (precondition), and the classification at the leaf node becomes the rule consequence [33]. Decision trees are easy to interpret, capable to work with missing values, categorical and continuous data, characteristics that make it interesting to use in the medical field.
- Random Forest, a meta-learner, comprising many individual trees, was developed to operate quickly over large datasets [34]. Each tree depends on the values of a random vector independently sampled and with the same distribution for all trees in the forest. For forests, the generalization error converges to a limit as the number of trees in the forest becomes larger [35]. The main advantages are related to its robustness to noise, and fast computation. However, results can be difficult to interpret, which counts as a disadvantage in the medical field.
- RIPPER is a rule-based induction algorithm. After producing a rule set for the class, each rule is reconsidered using reduced-error pruning, before proceeding to generate rules for the next class. RIPPER was evaluated through JRIP, that is, the implementation of RIPPER in Weka [30]. The main advantages of using this algorithm are a clear set of classification rules, and its speed.
- BayesNet is a Bayesian network based on probabilistic graphical models. It was developed under the presumption of nominal values (in case of numeric values, they are pre-discretized) and the absence of missing attributes [36]. Bayesian networks have had a significant impact on the modeling and on the analysis of the patient data. The major advantage is the easy interpretation of the results and robustness for dealing with missing data.

Evaluation experiments were performed using a 10-fold cross-validation setup. For each one of the 10 trials, the original dataset is partitioned into 10 subsets (folds) of equal size, P1,(…), P10. Each fold is used in turn as the test set, while the remaining nine subsets act as the training set. The global accuracy is the mean of the 10 trials accuracy. The Receiver Operating Characteristic (ROC) curve for each one of the classifiers was also plotted.

### 2.5. Model Evaluation

The validation was carried out using the previously developed models and was tested on the data from group III. The previously trained models were loaded to the class prediction of each pulse in this group, independently. The majority-voting system was used to merge the labels from each one of the algorithms in the final output.

The following nomenclature for predicted class labels was adopted: the label correlated to the APW patterns from group I (positive AIx values) was coded as A, while the “normal” APW patterns similar to those of group II (negative AIx values) were coded as B. Each subject can present both classes in its dataset, the final value being presented as a percentage of the class A and B within the set of the analyzed pulses.

## 3. Results

### 3.1. Patient Characteristics

The clinical characteristics of our study population are shown in Table 3. In group I, mean age is (59.05 ± 12.23) years, significantly greater than that of the healthy subjects (group II). The blood pressures are high (167.53 ± 13.04 mmHg and 102.10 ± 13.55 mmHg for SBP and DBP, respectively), relatively to the normal ranges, 120–129 mmHg for SBP and 80-84 mmHg for DBP [37], and significantly elevated compared to the group II. It was also verified that the Body Mass Index (BMI) is superior to the ideal recommendations (< 25 kg/m^2^) [37] and also significantly elevated considering the group II. The AIx values were elevated in these patients (9.57 ± 15.20%), comparatively with healthy subjects(–20.58 ± 20.03%), suggesting the presence of arterial stiffness. The pulses within this group were scored as class 1.

**Table 3:** Characteristics of subjects (n = 50).

**Variable**	**Group I**	**Group II**	**Group III**
Age (years)	59.05 ± 12.23 ^*^	24.40 ± 4.06	24.10 ± 2.81 ^†^
Gender (male/female)	9/11	11/9	1/9
Smoker (yes/no)	2/18	2/18	0/10
Weight (Kg)	73.87 ± 10.19	66.75 ± 10.72	58.00 ± 5.85 ^†^
Height (m)	1.63 ± 0.08 ^*^	1.71 ± 0.05	1.68 ± 0.07
BMI (kg/m^2^)	28.30 ± 5.50 ^*^	22.65 ± 2.80	20.56 ± 1.43 ^†^
SBP (mmHg)	167.53 ± 13.04 ^*^	110.50 ± 11.88	102.10 ± 13.35 ^†^
DBP (mmHg)	97.87 ± 11.24 ^*^	69.85 ± 10.47	72.90 ± 11.35 ^†^
HR (beats/min)	68.00 ± 6.72	67.80 ± 11.02	63.50 ± 8.89
AIx (%)	9.57 ± 15.20 ^*^	−20.58 ± 20.03	10.62 ± 7.86 ^*^^ †^

SBP-Systolic Blood Pressure, DBP-Diastolic Blood Pressure, BMI-Body Mass Index; Age, Weight, Height, BMI, SBP, DBP and HR are presented as *mean* ± *standard deviation*; ^† ^*p* < 0.01 compared with hypertensive subjects (group I), ^* ^*p* < 0.01 compared with healthy subjects (group II), by one-way ANOVA.

In group II, blood pressure ranges, are within normal values: 110.50 ± 11.88 mmHg for SBP, and 69.85 ± 10.47 mmHg for DBP. The subjects, in this group, present negative AIx values, as referred above, characteristic for healthy subjects. The pulses within this group were scored as class 2.

In group III, mean age is 24.10 ± 2.81, not significantly different from the values observed in healthy subjects (24.40 ± 4.06). No significant difference was noted between the Groups III and II in SBP and DBP values. However, the pulse waveforms are characterized by positive AIx values, 10.62 ± 7.86%, such as the values in group II, but significantly different.

### 3.2. Pulse Wave Analysis Pre-Processing

**Figure 2:**
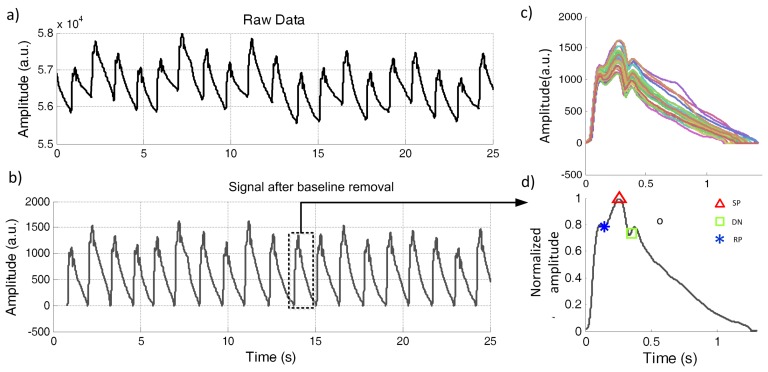
Pulse wave analysis, (**a**) raw data; (**b**) baseline removal; (**c**) pulse segmentation and (**d**) the prominent points marked in a pulse during the segmentation process.

Figure 2 depicts the effects of baseline removal and pulse segmentation procedures. A raw time signal segment of 25 seconds, corrupted by baseline fluctuation, is exhibited in Figure 2a and the baseline removal result is demonstrated in Figure 2b. Pulse-by-pulse analysis is shown in Figure 2c, where it is visible the pulse variability between pulses. In Figure 2d the prominent points (SP, Pi and DW) are pointed out.

### 3.3. Pulse Variance

**Figure 3:**
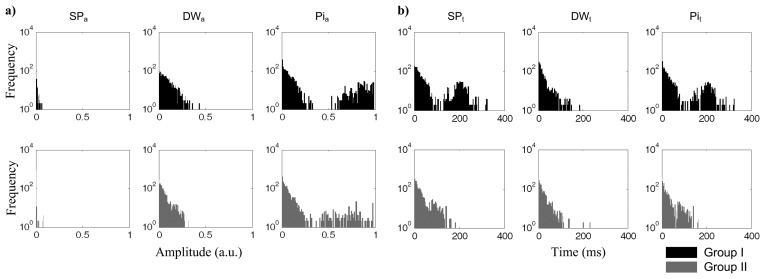
RMSSD successive differences measured for the SP, DW and Pi: (**a**) amplitude information; (**b**) time information.

The RMSSD values are plotted in Figure 3 where the variability associated to each one of the prominent points is assessed. Group I is represented in the upper row (black), and group II in the lower row (grey).

As SP amplitude (SP*_a_*) was previously normalized, during pre-processing, its relevance as far as the analysis is concerned is negligible. However, for SP*_t_*, it is possible to observe the presence of higher successive differences (RMSSD*_SPt_*) in group I. The similarities in both RMSSD*_DWt_* and RMSSD*_DWa_* parameters reveal the low predictive value of DW in this dataset. Concerning the inflection wave point time (Pi*_t_*), group I subjects show a significant increase in the distribution for RMSSD*_Pit_*.

**Figure 4:**
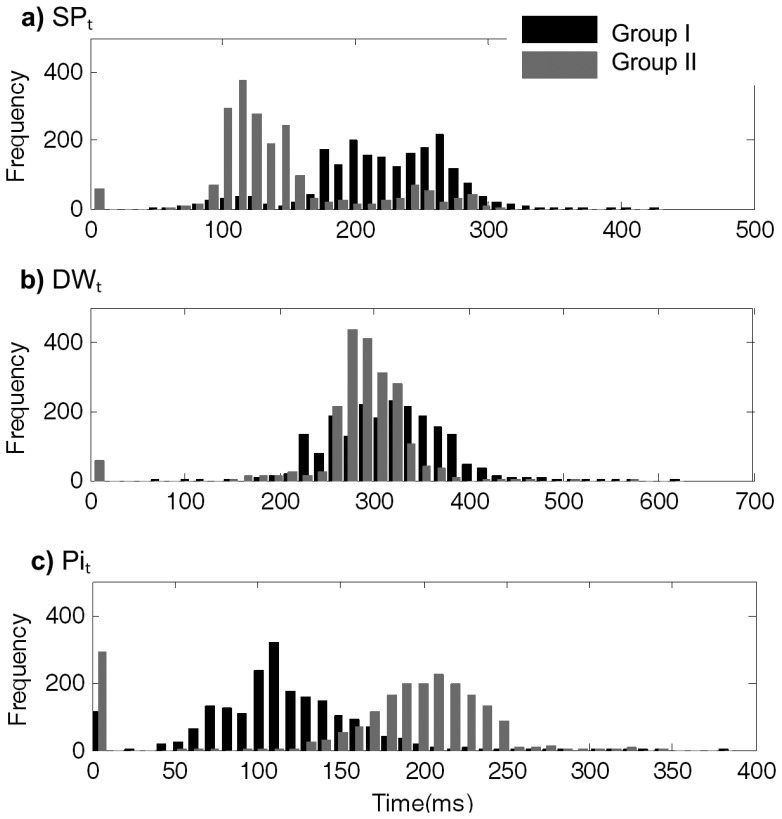
Arrival time histogram distributions for all of the studied prominent points (SP, DW and Pi).

Figure 4 shows the arrival time histogram distributions, within each segment, for SP*_t_*, DW*_t_* and Pi*_t_*. SP*_t_* occurs later in hypertensive group I, as depicted in Figure 4a, where arrival times assume the mean value of 219.99 ± 51.03 ms, while in group II this is at 144.12 ± 50.46 ms. DW*_t_* arrival time is quite similar for both groups: 313.52 ± 55.00 ms in group I and 291.84 ± 35.28 ms in group II (Figure 4b).

The Pi*_t_* occurs earlier in group I (118.94 ± 39.95 ms), as opposed to the arrival time in group II (200.46 ± 34.19 ms), as is visible in Figure 4c.

When the algorithm is not able to identify the prominent points due to morphological artifacts, or algorithm errors, a null timing is assumed and, consequently, the first histogram bin is incremented. These events occurred more frequently for Pi analysis, in healthy subjects, as is visible in the Figure 4b.

**Figure 5:**
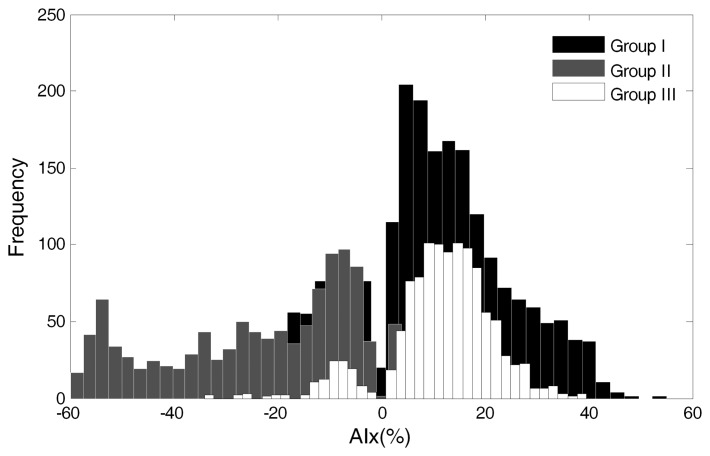
Augmentation index distribution for Groups I, II and III.

The magnitude of AIx was computed for each group (Figure 5). The positive AIx values from group I are visible in the black bars contrasting with the negative values of the grey bars of group II, as discussed in Section 3.1. In group III, the positive values are also predominant, as is visible in the white bars' histogram distribution. These values are further studied in Section 3.6.

### 3.4. Feature Selection

**Figure 6:**
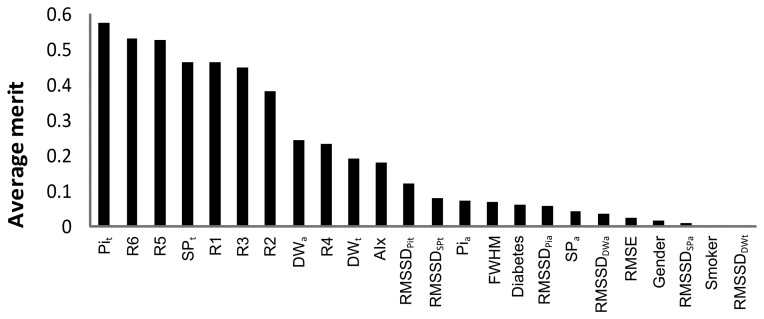
Predictive value analysis based on the average merit (numerical measurements taken from Weka 3-6-5 package).

The data from predictive value analysis are presented in Figure 6. Using the ranker method, InfoGainAttributeEval, we evaluated the importance criteria in the arterial stiffness determination (in reference to the subjects from group I). The parameters are ranked according to their predictive value that measures the degree of correlation within the class. This method individually assesses the predictive ability of each attribute and the degree of redundancy among them. Higher values are obtained for the ones that are highly correlated with class 1, but with low inter-parameter correlations. The Pi*_t_* and SP*_t_* time positions were revealed as the best predictors, as well as some ratios (R6, R1, R3 and R2) and the index (R5). This task was essential in discriminating the most interesting features to include in the classification model. The parameters with low merit (it was considered a threshold of 0.1) were discarded. The overall list of parameters selected and used in classification procedures include: Pi*_t_*, R6, R5, SP*_t_*, R1, R2, R3, DW*_a_*, R4, DW*_t_*, AIx and RMSSD*_Pit_*.

### 3.5. Classification

In this stage, the classifiers were evaluated using the accuracy (the percentage of correctly classified instances in a dataset) and the area under the ROC curve. The classification results, presented in Table 4, demonstrate the superior performance achieved by Random Forest. Classifiers within the 90%–95% accuracy gap are regarded as solid rules [38], which in this case represent three out of the four evaluated classifiers, the last one remaining very close to 90% (Table 4).

**Table 4:** Classifiers Selection.

**Classifier**	**Random Forest**	**J48**	**JRIP BayesNet**
Accuracy (%)	96.95	95.90	94.78 88.42

**Figure 7:**
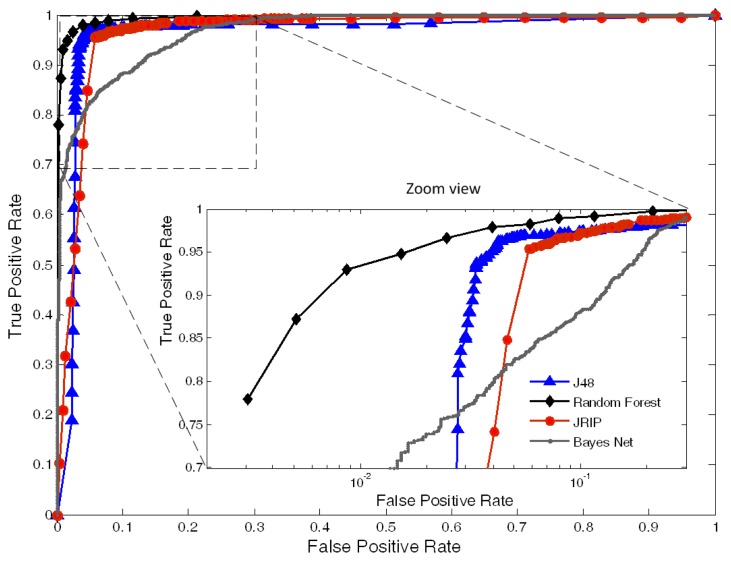
Semi-log ROC curves obtained with all classifiers (J48, Random Forest, JRIP, BayesNet).

The area under the curve (AUC) the ROC curves, corresponding to all the classifiers, are plotted in Figure 7. The area values, obtained for each one of the classifiers, were: 0.994 for Random Forest, 0.961 for J48, 0.965 for JRIP and 0.939 for BayesNet.

### 3.6. Model Evaluation

**Figure 8:**
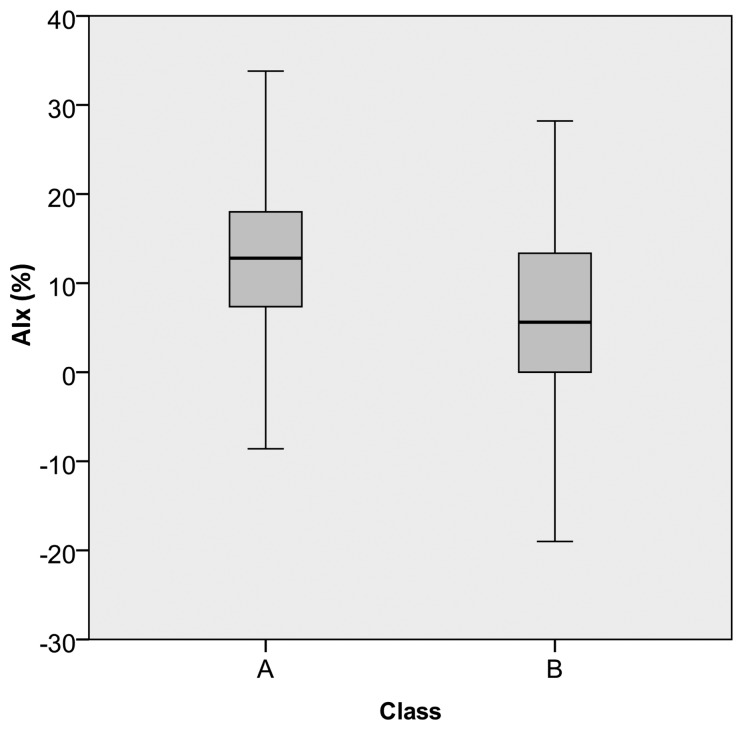
Boxplot of AIx distribution for classes A and B. The horizontal line within the box represents the median, the box represents the interquartile range (50% of the distribution) and the whiskers represent the range of values obtained for all subjects from group III in an overall set of 627 pulses.

The data prediction results are presented in Table 5. For each subject, the class was predicted using the previously developed models. All the pulses were considered, the final prediction thus resulting as a percentage of the number of class A and class B pulses, within the dataset. The information about AIx, being visible, the correlation between higher class A percentage and higher positive AIx values (Figure 8) are also included. For the pulses in class A, an average AIx value of 14.80 ± 8.60% was obtained and, for the class B group, a value of 4.10 ± 8.70% was observed.

**Table 5:** Data obtained from class prediction analysis and the AIx values (for each subject).

Subjects	Predicted class (%)		AIx (mean ± S.D.)
	A	B	
#1	87.13	12.87	19.05 ± 3.57
#2	86.09	13.91	12.28 ± 10.11
#3	76.00	24.00	16.15 ± 3.76
#4	65.53	34.47	7.13 ± 9.41
#5	62.98	37.02	10.70 ± 11.58
#6	61.85	38.15	12.46 ± 7.14
#7	61.04	38.96	11.50 ± 8.40
#8	60.66	39.34	9.89 ± 4.37
#9	54.87	45.13	6.63 ± 9.92
# 10	13.95	86.05	−3.33 ± 8.29

## 4. Discussion

This work introduces a data mining framework for APW assessment through the use of machine learning algorithms capable of dealing with vectorized morphological features. The validation of the proposed method was carried out based on a dataset of ten subjects. Although this is a limited number of subjects, which needs to be increased in further studies, we consider, for the time being, that the focus of this work should be on the methodology implementation rather than on the population validity scope.

Concerning the predictive value of the APW attributes (Figure 6) arrival time, Pi_t_ revealed to be the most significant in the “subjects”' discrimination. This relevance of Pi-related parameters can be explained by the known fact that arterial stiffness strongly correlates to its components (both in time, Pi*_t_*, and amplitude, Pi*_a_*). Clinically, this effect is usually quantified on the well-known AIx. Compared to the single AIx analysis, the proposed framework has the main advantage of being flexible in dealing with poor quality pulses, where pulse wave analysis can be difficult, or even impossible to perform. The difficulty in obtaining AIx values invariably occurs in cases where the inflection point is very close to (or buried in) the SP. In these cases, this approach is an effective way to sort out this ambiguity by the combination of the information coming out from several morphological parameters. Naturally, in the developed model, some degree of dependence on the accuracy of the inflection point determination remains. However, the interaction with other parameters, implied by the method, decreases this dependence to a point where prediction becomes reliably possible.

Naturally, systolic time (SP*_t_*) also appears as one of the key attributes in this dataset. Its importance is easily understood by the known relation with Pi. This effect is evident in Figure 4a, where, for hypertensive subjects, the higher SP*_t_* is visibly affected by the overlap of early reflected waves, that coming before SP*_t_* (Figure 4c).

The other indexes, R1, R2 and R3, still have considerable merit, but not as high as those of Pi and SP. However, they are quite important, because being Pi-independent parameters, they can turn robust the class prediction, whenever Pi is hidden in the APW and its parametrization is not achieved.

Dicrotic wave values (both DW*_t_* and DW*_a_*) changed less than expected, due to the absence of known cardiac valves complications in this dataset. Related to this fact, the low predictive value of the R4 ratio in the dataset was verified.

Through this study, we come to the conclusion that the combination of various classifiers should be used for the interpretation of APW data to avoid the specific limitations of single-parameter analysis. The final results clearly indicate that Random Forest performs better than the others, reaching an accuracy of 96.95%. Nevertheless, all the other algorithms have shown high classification results, especially in the case J48 and JRIP, where the results were high above 90.00% (95.90% and 94.78%, respectively). The less accurate was the BayesNet with 88.42%. One of the limitations of this analysis was the absence of studies for maximum accuracy levels. As the accuracy increases with the number of training samples, we are not aware if the maximum value has been achieved for each one of the classifiers. However, the levels of accuracy are high enough to assume that the maximum value was achieved during the training procedures. The future improvements of this method will include the study of the samples' optimal number in association with the learning time as an indication of optimizing the learning process. Results from the ROC curves, information about True Positive Rate (TPR) against false positive rate (FPR), confirm the good accuracy of the values. A very high TPR was achieved for Random Forest, J48 and JRIP contrasting with the BayesNet performance. These differences are clear in the zoom view in Figure 7, where the *x* axis is plotted in a logarithmic scale.

In the validation group, the subjects belonging to group III are young (24 years old, average) but also present positive AIx values, out of the normal range for this age [39], varying from −3.33% to 19.05%. With the application of the previous model, we have intended to discriminate patterns and relationships in the pulse morphology and the identification of risk value labels. These values were validated by the AIx, where it was verified a positive correlation between risk (class A in our dataset) and AIx positive values. The knowledge gained from this study provides more information than the one provided by typical pulse wave analysis techniques.

## 5. Conclusions

APW contains important physiological information easily identifiable by pulse wave analysis. The nature of arterial wave propagation of incident and of reflected waves plays a major role in the determination of important parameter indicators, which can serve as health status predictors of the cardiovascular system. The use of non-invasive methods for the identification of important APW patterns is a convenient and affordable method that can be applied during diagnostic trials.

The efficiency of a multi-parametric approach was demonstrated through the high scores achieved during the models development. The Random Forest revealed to be the most accurate classifier. Among the studied parameters the higher predictive values were achieved by Pit and Pi_a_, considering the fact that arterial stiffness strongly correlates to Pi.

The future improvements of this approach should focus on the exploitation of other machine learning algorithms and the expansion of the number of parameters. It may include variants of Artificial Neural Networks (ANN) and Support Vector Machines (SVM) that can improve the accuracy due to its capability in dealing with non-categorical features. We also consider that the inclusion of data from biochemical analysis and from cardiac equipment setups can provide a more helpful tool, able to correlate the information retrieved from APW with other cardiac disorders. Another promising approach in this related field is the use of the information of total acquisition time (without pulse segmentation), allowing the analysis of dynamic information.

The healthcare public and private system faces the challenge of an increasingly aging population and the escalation of medical costs. Therefore, the development of techniques that allow for earlier identification of subjects at risk, by the incorporation of personalized, predictive and preventive methodologies, are essential.
